# Prevalence and predictors of low bone mineral density in Myasthenia gravis

**DOI:** 10.1055/s-0046-1824431

**Published:** 2026-07-03

**Authors:** Renata Dal-Prá Ducci, Lucas Ferreira Santana, Claudia Kamoi Kay, Raphael Henrique Déa Cirino, Paulo José Lorenzoni, Otto Jesus Hernandez Fustes, Paula Raquel Do Vale Pascoal Rodrigues, Carolina Aguiar Moreira, Rosana Hermínia Scola

**Affiliations:** 1 Universidade Federal do Paraná, Hospital de Clínicas, Departamento de Medicina Interna, Divisão de Neurologia, Curitiba PR, Brazil.; 2 Universidade Federal do Paraná, Hospital de Clínicas, Departamento de Medicina Interna, Curitiba PR, Brazil.; 3 Universidade Federal do Paraná, Hospital de Clínicas, Departamento de Medicina Interna, Divisão de Endocrinologia, Curitiba PR, Brazil.

**Keywords:** Myasthenia Gravis, Densitometry, Osteoporosis, Bone Density, Bone Diseases

## Abstract

**Background:**

Myasthenia gravis (MG) is an autoimmune disease affecting the postsynaptic neuromuscular junction. Treatment often includes corticosteroids, which may reduce bone mass.

**Objective:**

To evaluate the prevalence and predictors of low bone mineral density (BMD) in MG patients.

**Methods:**

A cross-sectional study included patients over 18 years with MG followed at the Neuromuscular Diseases Service, Hospital de Clínicas, Universidade Federal do Paraná (2018–2022). Data were obtained through clinical evaluation, medical records, MG Composite Scale, and Quantitative MG Test. Lumbar spine and proximal femur BMD were assessed using a GE Lunar densitometer, with T or Z-scores according to age/menopausal status. Logistic regression identified factors independently associated with BMD alterations.

**Results:**

In total, 92 patients (64.1% female, mean age: 51.3 years) were included, of whom 79.3% had early-onset MG. Past or present corticosteroid use were reported in 94.5 and 45.6%, respectively. Only 27.6% practiced regular physical activity, though most had adequate calcium intake. There were six (7.8%) patients with fragility fractures, and ⅕ were at high fracture risk according to the FRAX results. We found that BMD alterations occurred in 43.5% of patients. No significant association was found with corticosteroid use or MG severity. Age was independently associated with BMD (OR = 1.06, 95% CI: 1.03–1.09, *p* = 0.0005). A cutoff age ≥ 49 years yielded 82.5% sensitivity, 63.5% specificity, and an area under the ROC curve of 0.72 (95% CI: 0.61–0.83).

**Conclusion:**

Reduced BMD is frequent in MG, highlighting the importance of screening and prevention of low bone mass, osteoporosis, and fragility fractures, particularly in patients over 49 years, regardless of corticosteroid use or MG severity.

## INTRODUCTION

Myasthenia gravis (MG) is an autoimmune disease that affects the postsynaptic portion of the neuromuscular junction. 1 Clinically, it is characterized by muscle weakness, predominantly affecting proximal, bulbar, and/or ocular muscles, with fatigability and fluctuation. Furthermore, the diagnosis can be made by the combination of clinical, specific autoantibodies, and electrophysiological tests. 1

It is estimated that the prevalence of MG in the Latin American population is fewer than 100 per 100 thousand inhabitants, though Brazilian-specific data are lacking. 2 The incidence of MG follows a bimodal distribution, with a peak around 30 years of age, predominantly affecting female subjects, and a second peak after 50 years of age, with a slight predominance in male subjects. 3 For this reason, MG patients are classified as having early-onset MG (before 50 years) or late-onset MG (after 50 years). 4

The treatment of MG includes the use of an acetylcholinesterase inhibitor often in combination with immunosuppressive therapies such as corticosteroids or other medications like azathioprine, cyclosporine, and mycophenolate mofetil. 3 Despite the frequent use of therapies that negatively affect bone tissue, 5 6 the association between MG and the risk of fractures or reduced bone mass is controversial. 7 8 9 Regardless, identification and calculation of risk factors for fragility fractures by FRAX (Osteoporosis Research Ltd.) and measurement of bone mineral density (BMD) using dual-energy X-ray absorptiometry (DXA) are recommended, as well as preventive strategies to avoid bone loss and fractures. 10

The objective of this study is to establish the clinical-epidemiological profile of MG patients followed at a tertiary hospital and assess the prevalence and predictors of reduced BMD in these individuals.

## METHODS

**Figure 1:**
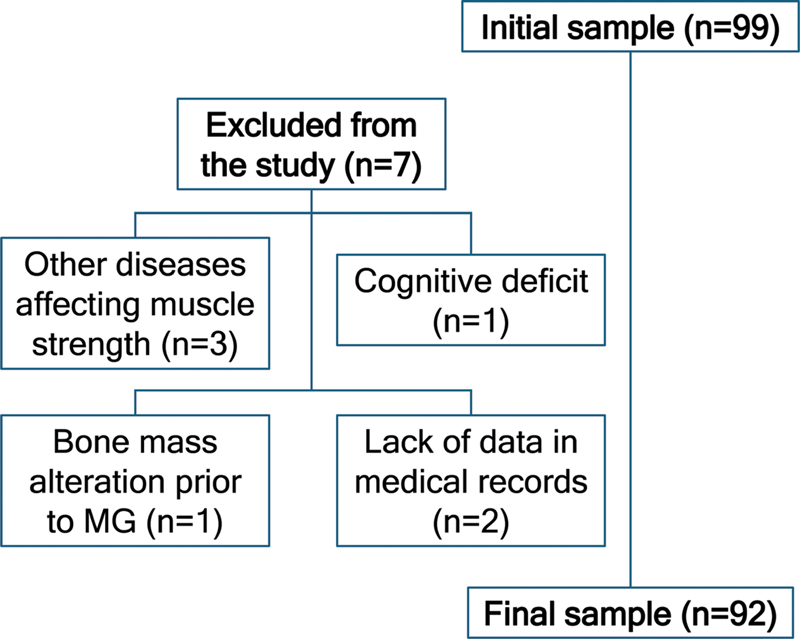
Flow diagram of the study sample selection.

The current is a cross-sectional observational study approved by the institutional Ethics on Research Committee. The study included individuals over 18-years-old, with a confirmed diagnosis of MG through clinical features, serum level of autoantibodies (anti-AchR, anti-Musk), and electrophysiologic tests according to previous recommendations, 1 who were under follow-up at a neuromuscular disease referral center in Brazil between 2018 and 2022, and whose DXA was performed less than 1 year before the date of inclusion. Individuals with other health conditions that could affect muscle strength, as well as those with a prior diagnosis of bone mass alterations before their MG diagnosis or secondary osteoporosis were excluded, as shown in Figure 1 . Patients unable to comprehend the proposed tests or who did not sign the Informed Consent Form were also excluded.

Clinical data were collected through medical record reviews and interviews, during which the following clinically validated scales for Brazilian Portuguese language were applied: the myasthenia gravis quality of life questionnaire (MGQOL-15), 11 the myasthenia gravis composite scale (MGCS), 12 and the quantitative myasthenia gravis test (QMG). 13 Considering the age of symptom onset, patients were classified as having early onset MG or late onset MG. To assess the participants' exposure to corticosteroids, the cumulative corticosteroid dose was calculated based on medical records, considering the dose used multiplied by the number of days of use within the year prior to the DXA exam. 6

The BMD measurements of lumbar spine and proximal femur were performed at the Bone Densitometry Service of our institution using a GE-Lunar (GE HealthCare) densitometer with minimal variance significative of 0.025 g/cm for lumbar spine and 0.028 g/cm ^2^ for proximal femur. The DXA evaluates bone mass in grams per square centimeter and is interpreted using T-scores or Z-scores, depending on age and menopausal status. T-score compares an individual's BMD to that of a healthy young adult of the same sex and was used for postmenopausal women and men over 50-years-old. The Z-score compares the BMD to that of a healthy individual of the same age and was used for premenopausal women and men under 50-years-old. 5 Furthermore, T-score values up to -1 were considered normal, while those between -1 and -2.5 indicated osteopenia, and a value of -2.5 or lower indicated osteoporosis. A Z-score lower than -2 indicated low bone mass. 10 Reduced BMD was considered if the patient fulfilled criteria for low bone mass, osteopenia, or osteoporosis.

For statistical analysis, mean and standard deviation (SD) values were used for normally distributed continuous variables, while median and interquartile range (IQR) values were applied for non-normally distributed continuous variables. Frequencies and percentages were reported for categorical variables. Comparisons of clinical variables between MG patients with and without BMD alterations were performed using Student's *t* -test, Wilcoxon's rank sum test, and Fisher's exact test. A stepwise logistic regression model with *p* < 0.05 inclusion criteria was applied to evaluate parameters independently associated with BMD alterations. All variables with *p* < 0.1 were included in the model. A sensitivity and specificity analysis with determination of the best cut-off point for detecting BMD alterations was performed using the receiver operating characteristic (ROC) curve. Significant associations were considered when *p-value* was minor than 0.05. Statistical analyses were performed using the software Stata for Windows (StataCorp LLC.), version 18.5.

## RESULTS

The study sample consisted of 92 individuals, 64.1% of whom were female ( *n* = 59), with a mean age of 51.3 ± 15.7 years. Based on the age of symptom onset, 79.3% ( *n* = 73) were classified as having early-onset MG. The median age of symptom onset and duration of MG were 29.5 (IQR: 20.0–47.2) years and 14 (IQR 6.6–24.3) years, respectively. Regarding the autoantibody profile, 77.1% tested positive for anti-AChR antibodies and 5.4% tested positive for anti-MuSK antibodies. The main comorbidities observed in the sample were obesity (58.6%), systemic arterial hypertension (36.9%) and diabetes (22.8%), as presented in Table 1 . As for smoking habits, only 2 individuals (2.6%) reported current smoking, while 18 (23.6%) reported being former smokers.

**Table 1:** Clinical profile of individuals with MG

Variable		MG patients
Female, n (%)		59 (64.1)
Age in years, mean ± SD		51.3 ± 15.7
Age of onset in years, median (IQR)		29.5 (20.0–47.5)
MG classification by age of onset, n (%)		Early-onset: 73 (79.3) Late-onset: 19 (20.7)
Duration of disease in years, median (IQR)		14 (6.8–24.3)
BMI, kg/m ^2^ , mean ± SD		27.5 ± 5.6
Anti-AchR serum dosage, n (%)		Performed: 91 (98.8) Positive: 71 (77.1)
Anti-MuSK serum dosage, n (%)		Performed: 21 (22.7) Positive: 5 (5.4)
MGCS score, median (IQR)		3 (1–6)
QMG score, median (IQR)		8 (4–13)
MGQOL score, median (IQR)		9 (3–25)
MGFA classification, n (%)	Asymptomatic	20 (21.7)
	1	15 (16.3)
	2A	33 (35.8)
	2B	2 (2.1)
	3A	15 (16.3)
	3B	5 (5.4)
	4	2 (2.1)
Comorbidities, n (%)	Obesity	54 (57.4)
	Hypertension	34 (36.9)
	Diabetes mellitus	21 (22.8)
	Psychiatric disorder	19 (20.6)
	Dyslipidemia	16 (17.4)
	Hypothyroidism	14 (16.3)
	Fibromyalgia	4 (4.3)
	Cataract	4 (4.3)
	Others	29 (31.5)

Abbreviations: BMI, body mass index; IQR, interquartile range; MG, myasthenia gravis; MGCS, myasthenia gravis composite scale; MGQOL-15, myasthenia gravis quality of life questionnaire; QMG, quantitative myasthenia gravis; SD, standard deviation.

Considering the MG specific clinical scales, the median score on the MGCS was 3 (IQR: 1–6), 8 (IQR: 4–13) on the QMG, and 9 (IQR: 3–25) on the MGQOL. The medications used by the patients are detailed in Table 2 . In the entire group, 94.5% had used glucocorticoids at some point during their MG treatment, with a median prednisone cumulative dose of 1,350 (IQR: 0–5,655) mg/year. At the time of the study, 45.6% ( *n* = 42) of the patients were using prednisone, with a median dose of 15 (IQR: 5–20) mg, and 33 of these individuals (78.6%) were receiving calcium and vitamin D supplementation. Other medications classically associated with increased risk of osteopenia or osteoporosis, along with their median, maximum, and current doses, are presented in Table 2 .

**Table 2:** Current and past medications used by individuals with MG

Medication		
Prednisone	Lifetime use, n (%)	87 (94.5)
	Duration of use (years), median (IQR)	4 (2–11.25)
	Maximum dose (mg/day), mean ± SD	46.7 (24.1)
	Current use, n (%)	42 (45.6)
	Current dose (mg/day), median (IQR)	17.5 (5–20)
	Cumulative dose (mg/year), median (IQR)	1,350.0 (0–5,655.0)
Azathioprine	Lifetime use, n (%)	62 (66.3)
	Duration of use (years), median (IQR)	4.0 (1.3–9.8)
	Maximum dose (mg/day), mean ± SD	145.0 ± 42.3
	Current use, n (%)	47 (51.0)
	Current dose (mg/day), median (IQR)	125.0 (100.0–150.0)
Cyclosporine	Lifetime use, n (%)	12 (13.0)
	Duration of use (years), median (IQR)	7.0 (4.0–8.0)
	Maximum dose (mg/day), mean ± SD	197.5 ± 74.9
	Current use, n (%)	10 (10.8)
	Current dose (mg/day), median (IQR)	100.0 (75.0–225.0)
Mycophenolate	Lifetime use, n (%)	7 (7.6)
	Duration of use (years), median (IQR)	4.0 (3.0–4.0)
	Maximum dose (mg/day), mean ± SD	1,583.0 ± 273.8
	Current use, n (%)	7 (7.6)
	Current dose (mg/day), median (IQR)	1,000.0 (1,000.0–1,500.0)
Dietary supplements	Calcium supplementation, n (%)	49 (53.2)
	Vitamin D supplementation, n (%)	54 (58.7)

Abbreviations: IQR, interquartile range; MG, myasthenia gravis; SD, standard deviation.

Regarding factors affecting bone health, considering the whole sample, supplementation of calcium was provided to 53.2% ( *n* = 49) and of vitamin D to 58.6% ( *n* = 54) individuals. Adequate daily calcium intake, including both dietary sources and supplements, was observed in 63% of the sample. However, when considering dietary sources alone, 22 individuals (23.9%) met the recommended calcium intake. 5

Additionally, 21 individuals (27.6%) reported engaging in regular physical exercise. The median exercise duration was 40 minutes (IQR: 30–60) with a frequency of three to five times per week in most cases, mainly involving aerobic activities. Only 2 individuals reported routinely performing resistance exercises.

A history of fragility fractures, defined as a fracture caused by minimal or low-energy trauma, 10 was found in 6 individuals, of whom 4 were female, with a median age of 56.5 (IQR: 48.3–63.3) years, a median age of MG duration of 19.5 (IQR: 12.3–28.3) years, and 2 cases still using corticosteroids. Furthermore, the risk of fractures, as calculated by FRAX, was high for major fractures in 16 individuals (21.3%) and for hip fractures in 16 (21.3%). In the sample studied, 43.5% ( *n* = 40) of individuals showed some alteration in BMD, with 5.4% ( *n* = 5) presenting low bone mass, 30.4% ( *n* = 28) osteopenia and 7.6% ( *n* = 7) osteoporosis. Grouping men over 50 years of age and postmenopausal women, 35 (70%) showed changes in BMD: 7 out of 50 (14%) had osteoporosis and 28 out of 50 (56%) had osteopenia, whereas when grouping men under 50 years of age and premenopausal women, only 5 (11.9%) showed low bone mass. At the time of the evaluation, all patients diagnosed with osteoporosis were using bisphosphonates.

**Figure 2:**
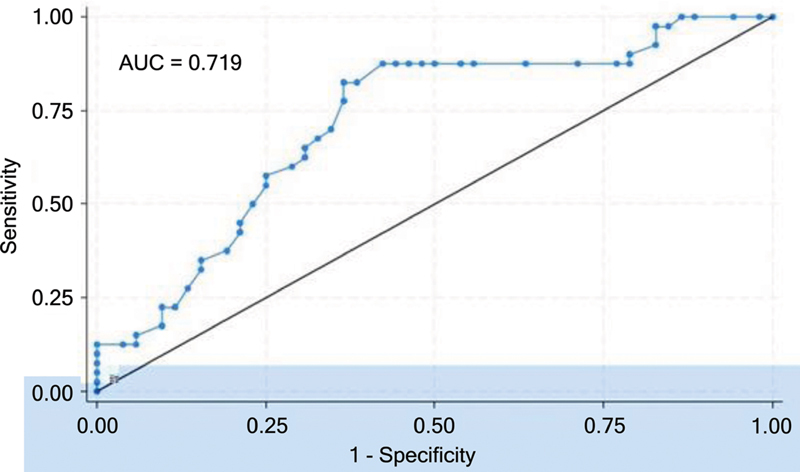
Abbreviations: AUC, area under the ROC curve; BMD, bone mineral density; ROC, receiver operating characteristic. Most accurate cutoff value for age to detect low BMD, according to the ROC curve analysis.

No significant association between reduced BMD and corticosteroid use among patients with MG was observed ( *p* = 0.401), neither between BMD alterations and MG severity, as measured by the QMG scale ( *p* = 0.9 *37* ) and MGCS ( *p* = 0.99), as shown in Table 3 . Other associations with alterations in BMD were tested, but except for the age ( *p* = 0.001), none had statistically significant results, as shown in Table 3 . In the multivariate regression model, the age remained independently associated with BMD alterations (OR = 1.06, 95%CI: 1.03–1.09, *p* = 0.0005). Through the ROC curve analysis, the most accurate cutoff value for age to detect low BMD was 49 years, with 82.5% sensitivity, 63.5% specificity and an area under the curve (AUC) of 0.7195 (95%CI: 0.612–0.826), as shown in Figure 2 .

**Table 3:** Associations between clinical variables and BMD alteration in MG patients

Variable	BMD alteration group (n = 40)	Normal BMD group (n = 52)	*p* -value
Age, years	58.2 ± 14.4	46.0 ± 14.7	0.001*
Female, n (%)	26 (65.0)	33 (63.5)	1.000**
Early-onset MG, n (%)	28 (70.0)	45 (86.5)	0.070**
Age of onset, years	38.0 (19.5–54.0)	25.0 (20.5–38.0)	0.059***
MG duration, years	14.5 (8.0–28.5)	12.5 (5.5–24.0)	0.187***
MGCS	3 (1–6)	3 (1–6.5)	0.990***
QMG	8 (4–13)	7.5 (4–13.5)	0.937***
MGQOL15	9 (3.5–25.5)	9 (1.5–24)	0.624***
Corticosteroid current use, n (%)	16 (40.0)	26 (50.0)	0.401***
Time through corticosteroid usage, years	6.0 (2.0–12.5)	4.0 (2.0–10.5)	0.280***
Current corticosteroid dosage, mg/day	20.0 (7.5–25.0)	15.0 (5.0–20.0)	0.345***
Cumulative corticosteroid dosage, mg/year	0 (0–4,912.5)	1,987.5 (0–6,190.0)	0.166***
Azathioprine current use, n (%)	20 (50.0)	27 (51.9)	1.000**
Cyclosporine current use, n (%)	3 (7.5)	7 (13.5)	0.505**
Mycophenolate current use, n (%)	2 (5.0)	5 (9.6)	0.695**
Adequate calcium intake, n (%)	29 (72.5)	29 (55.8)	0.128**
BMI, kg/m ^2^	26.8 ± 5.6	28.1 ± 5.7	0.270*
Physical exercise practice, n (%)	7 (17.5)	14 (26.9)	0.311**

Abbreviations: BMD, bone mineral density; BMI, body mass index; MG, myasthenia gravis; MGCS, myasthenia gravis composite scale; MGQOL-15, myasthenia gravis quality of life questionnaire; QMG, quantitative myasthenia gravis.

Notes: *Mean (standard deviation) – Student's t test. **Fisher's exact test. ***Median (IQR) – Wilcoxon rank sum test.

## DISCUSSION

This study found a high prevalence of low BMD in individuals with MG who were followed at a neuromuscular disease reference center. Most of these patients had received high doses of corticosteroids over their lifetime, with nearly half still using them. Almost ⅓ of the patients were at high risk for fractures according to the FRAX tool. Although, more than half of patients reported adequate calcium intake, other important lifestyle habits for bone health, such as regular physical exercise, were absent in more than half of the patients. In our sample, age was a strong predictor for BMD alterations.

The study sample consisted of 59 women out of 92 total participants, with an approximate 2:1 ratio, which differs from the ratio reported in other global epidemiological studies. 14 However, it was similar to findings from an epidemiological study conducted in the state of Ceará, Brazil 15 and from the study by Wakata et al., 16 which demonstrated a higher prevalence of MG in women. The higher prevalence of early-onset MG in this study's sample could explain the greater proportion of females, as this age group is known to have a higher female prevalence. 3 The remaining characteristics of our sample are consistent with other studies. 2 3 Furthermore, it's important to emphasize that although the average severity of the disease was classified as mild, this was assessed only once. It is likely that the true burden of the disease is also reflected by the median symptom duration, which in our study was 14 years—highlighting the chronic nature of this condition, often requiring sustained exposure to corticosteroids, as observed in our sample. Besides that, in general, MG tends to have better control after 2 years from the onset of symptoms, 17 which is compatible with the duration of illness in our sample.

As for physical activity, only a minority engaged in physical exercise (27%). Among those who reported engaging in some type of exercising, only 2 practiced resistance training, which involves prolonged muscle contraction, a nonpharmacological intervention recommended for preventing bone mass loss, regardless of age. 18 Consequently, despite accumulating several risk factors, MG patients do not participate in some of the activities recommended for the treatment and/or prevention of osteometabolic diseases, either due to sociocultural factors or the morbidity caused by the disease itself.

The Position Statement of the Latin American Federation of Endocrinology on Osteoporosis and the International Consensus Guidance for the Management of Glucocorticoid Related Complications in Neuromuscular Disease recommend a daily intake of 800 to 1,200 mg of calcium as a non-pharmacological measure to reduce risk of osteoporosis and fractures, equivalent to 3 to 4 servings of milk or dairy products per day. 10 19 20 21 Furthermore, the association with vitamin D is mandatory, since it is responsible for calcium absorption in the intestine. Although the majority of patients consumed only one to two servings of those products daily, most of them achieved adequate calcium intake through supplementation during follow-up with their neurologist, reinforcing the importance of assessing the need for supplementation in these patients.

The chronic glucocorticoid treatment combined with these other risk factors underscore the necessity of screening for low BMD and implementing appropriate preventive measures in individuals with. 7 22 The risk of fractures should be assessed as soon as corticosteroid therapy exceeding 2.5 mg/day is initiated for more than 3 months, even considering a bigger bone mass loss in the first months of treatment. 6 21 A DXA scan is necessary for all these individuals, and fracture risk should be calculated using the FRAX tool if they are over 40-years-old. 23 24 Subsequently, patients on chronic corticosteroid use should undergo the same evaluation every 1 to 2 years. 6

Prevention of low BMD and fractures should include nonpharmacological measures, such as increasing calcium and vitamin D-rich foods in the diet, and, when dietary intake is insufficient, prescription of supplementation. 22 25 Furthermore, it is crucial to promote routinely physical exercise, especially resistance training, 10 which was not frequent in our sample. We also encourage minimizing MG patients' exposure to corticosteroids and, when possible, prescribe corticosteroid-sparing therapies.

In our study, over 1/5 of the patients were at high risk for major osteoporotic fractures, as estimated by FRAX (Brazil version). This tool can estimate the risk of osteoporosis and fractures in patients at increased risk for these conditions, 23 24 and it is particularly important in areas where access to BMD testing is limited, as it enables prophylactic interventions. 25 Specifically, a Japanese cohort study showed that individuals with MG and a higher FRAX score had a greater incidence of fragility fractures over 10 years, being an even stronger risk predictor than the T-score alone. 26 27 In our sample, 7.8% of the patients had a history of fragility fractures. It is known that the occurrence of fragility fractures is associated with greater morbidity and can be a cause of pain and loss of function. 10

Additionally, this study revealed a high prevalence of low BMD. The prevalence of osteoporosis in this study (7.6%) was lower than the average expected in the Americas (12.4%) and globally (18.3%), as reported by a 2021 meta-analysis, 26 but similar to the study by Wakata et al. 16 However, when considering male individuals older than 50 years and women in postmenopause, this prevalence increases to 14%. The presence of BMD alterations was statistically associated with aging, in agreement with previous studies. 5 6 10 Our study found a moderate accuracy of age over 49 years for detection of BMD alterations. This reinforces the need for vigilance of BMD alterations in patients with MG, mainly in individuals older than 49 years, regardless of the corticosteroid use status, and considering screening with BMD evaluation earlier than usually recommended. 5 10

Some studies indicate that MG itself may contribute to the risk of osteopenia and osteoporosis. 7 16 21 28 29 Despite this, the present study was not able to find a significant relationship between clinical condition—assessed by the QMG and MGCS scales—and BMD alterations, as well as the study by Wakata et al., 16 which did not demonstrate a correlation between the duration of MG and changes in BMD. 26

Most individuals used or were still using glucocorticoids. The cumulative prednisone dose was analyzed, as it varies significantly among patients over a year, with the cumulative dose being a more accurate marker of actual exposure. 30 It should be noted that the cumulative dose was measured by the sum of the doses used over the last 365 days before the study's interview. 30 It was found that over 70% of the patients were using daily doses related to increased risk for osteoporosis and other bone mass alterations. 21 30 In contrast, the present study found no association between corticosteroid use and BMD alterations, similar to Safipour et al. 29 This finding may be due to an underpowered sample size or due to the action of the corticosteroids modifying the bone tissue microarchitecture rather than just reducing bone mineral density. 31

Among the study's limitations, it was conducted in a single reference center with a small sample size, which limits the generalizability of the results and the statistical power to detect significant differences between the BMD groups. Another limitation is the cross-sectional nature of the study, which precludes establishing causal relationships and does not allow us to assess the real severity of the disease, since it could be well controlled by the time of the assessment, masking its lifetime burden. Also, due to the study design, the lack of a control group (e.g., non-MG corticosteroid users or MG patients not on corticosteroids) makes it difficult to isolate the MG-specific contribution to bone changes.

On the other hand, it is important to note that this study contributes to characterize the clinical and bone profiles of MG patients. Furthermore, we revealed a high prevalence of low bone mass in a sample of patients with a confirmed diagnosis of MG who are followed at a neuromuscular diseases reference center, regardless of the use of corticosteroid therapy or the severity of the underlying disease. Additionally, it was possible to identify an age cutoff point with moderate accuracy for the detection of BMD alterations in MG patients.

In conclusion, a high prevalence of low BMD was observed in patients with MG followed in a tertiary hospital. Therefore, it is essential to evaluate bone health in all patients with MG, independently of severity and the use of corticosteroids, mainly in individuals over 49-years-old, and address preventive approaches, including calcium and vitamin D intake along with exercises, to reduce bone loss and fragility fractures.
